# The London Ambulance Question: Its History and Present Position

**Published:** 1914-01-10

**Authors:** 


### THE LONDON AMBULANCE QUESTION.

### Its History and Present Position.

It was in 1902 that Sir William Collins first presented a report to the London County Council on an ambulance service for street accidents in London. The report showed that London was far behind other large towns, at home and abroad, in regard to the methods of dealing with accidents and other casualties in streets and public places. Hand litters were employed, the telephone was not utilised, cabs, vans, and barrows were often used to convey persons seriously injured to hospitals. The report devised a scheme for rapid ambulances electrically propelled, summonable by telephone, for dealing with these cases. In 1906 the County Council inserted clauses in its General Powers Bill with a view to inaugurate such service in London. The estimated capital cost was £13,000, and the annual maintenance £9,600.

These clauses passed the Commons, but were thrown out in the Lords. A Departmental Committee of the Home Office was then set up, and a fresh inquiry embarked upon. The Committee consisted of Sir Kenelm Digby (chairman), the late Lord Stamford, and Sir William Collins, M.P. Meanwhile the City Corporation, which had opposed the powers sought by the County Council, adopted their scheme, and have since most successfully worked an electric ambulance service summonable by telephone in the City area. The Committee reported in March 1909, and pronounced the City's arrangements "very satisfactory," and unanimously found that "it has been abundantly shown that the present system (outside the City) is gravely defective, and results in much preventible detriment and suffering by reason of the transport, by unsuitable means, of persons who have been injured or taken ill in the streets or other public places."

Interrogated by Sir William Collins in April 1909, the Home Secretary (Mr. Gladstone) could hold out no hope of legislation that year. On June 14, in a debate on supply, the Home Secretary encouraged Sir William Collins to proceed with a Bill he had introduced to enable the London County Council to supply, or aid the supply, of an ambulance service for the County of London. When the Metropolitan Ambulances Bill was in Committee on August 17, the Home Secretary said: "The Metropolitan Asylums Board did very excellent work, but to invest it with the whole ambulance work of London would be to give it duties and authority for which it was not constituted, and for which it was not specially designed. There was not, he thought, the smallest chance of getting the House of Commons to accept the Asylums Board as the authority, and if so, to press forward such a proposal would cause dangerous delay. This was a matter of urgency." The Committee endorsed this view by 28 to 1 on a division. In the Lords on September 14, 1909, Earl Beauchamp said: "The position of the Home Office is this—there is some fear that the public at any rate would think that the ambulances of the Board are liable to infection. . . . The London County Council would be able to use a certain amount of land in their possession, and, altogether, in the opinion of the Home Office, they are the proper authority to undertake the work." The Bill received the Royal Assent on October 20, 1909.

Four years have elapsed, and the London County Council have done little beyond inquire during that time. True, they have during the current year put the Act of 1909 into operation so far as to maintain a motor ambulance presented by the Grand Duke Michael of Russia to the Hampstead and North-West London Hospitals, and which is available for street accidents in the North-Western district. They have endeavoured to shuffle off their responsiblities on to the Metropolitan Asylums Board, the Borough Councils, and Boards of Guardians, and in their General Powers Bill of last session sought enabling powers for these bodies to press any ambulances, cabs, or stretchers they possess into the service for street accidents, while the financial responsibility would, under the Act of 1909, rest exclusively with the County Council.

Sir William Collins, in a memorandum which he added to the report of the Departmental Committee, disavowed "any desire that any difference of opinion as to the body which should be authorised to establish an improved ambulance service for London should in any way prejudice or impede the prompt inauguration of such service." He, however, pointed out that "the notion that economy would result from imposing these new duties upon the Asylums Board will, on examination, prove to be illusory. Such notion appears to be based on the assumption that in some way or other the existing stations of the Board, which cost some £120,000, together with their plant and staff, could in the main be made to serve the purposes of the street service, and on the further assumption that the charges made by them for 'non-street cases' would largely cover the cost of the street service. Unfortunately neither assumption has proved to be well founded. The report shows that it will be impracticable to make a charge for the use of the ambulance for 'street cases.' . . . Far more important, however, is the fact that while the Asylums Board stations are admirably situated for the infectious service, which is so satisfactorily rendered, they are, by reason of their disposition, mainly in the outer zone of London, ill-arranged for the purposes of a 'street' service. It was insisted by Mr. Mann, the able Clerk of the Board, that in the event of this unsolicited duty being cast upon the managers, it would be necessary, even if their existing stations were utilised at all, to establish not fewer than ten additional self-contained stations, somewhat after the model of the City station, in central parts of London. Moreover, it is regarded as imperative that if the Board's existing infectious stations are utilised at all there must be absolute and complete physical separation both of *matériel* and *personnel* between the infectious and the street service. It is further admitted that the greater portion of the new street service would, in all probability, be performed by the new centrally situated self-contained stations which must be provided. It therefore appears to be incurring uncalled-for risk, and introducing unnecessary complication, with little, if any, saving of expenditure, to set up any of the 'street' ambulance stations on the sites at present occupied by the infectious services of the Asylums Board. . . . Lastly, the Metropolitan Asylums Board would apparently not have, as the London County Council has, sites or buildings ready at hand in the centre of London for the new street stations required."

The scheme which the Metropolitan Asylums Board recently formulated for the County Council, and which the latter alone can pay for, was practically a reproduction of the schemes of 1902 and 1905 formulated by the Progressives. The capital cost, £18,680, and maintenance, £17,860 a year, is not more economical, but more costly than that which Sir William Collins designed. The London County Council remains the only local authority in London which has sought, and which possesses, power to establish and maintain out of the rates a street ambulance service. Here, again, the authority which pays the piper should call the tune.

The County Council at their last meeting before Christmas declined with thanks the scheme of the Asylums Board which they had themselves invited. Major Gray, the Chairman of the General Purposes Committee, admitted that for the Council to pay for a scheme worked by the Board was administratively impossible, and that new stations with motor ambulances would have to be provided in the danger areas, under the Act of 1909.

